# Highly Efficient Cd^2+^ Removal Using Tobermorite with pH Self-Adjustment Ability from Aqueous Solution

**DOI:** 10.3390/ma16031314

**Published:** 2023-02-03

**Authors:** Juan Qin, Sujuan Yuan, Mauricio Córdova-Udaeta, Keishi Oyama, Chiharu Tokoro

**Affiliations:** 1 School of Chemistry and Chemical Engineering, Nantong University, Nantong 226019, China; 2 Faculty of Science and Engineering, Waseda University, 3-4-1 Okubo, Shinjuku-ku, Tokyo 169-8555, Japan; 3 Faculty of Engineering, University of Tokyo, 7-3-1 Hongo, Bunkyo-ku, Tokyo 113-8656, Japan

**Keywords:** tobermorite, pH self-adjustment, cadmium, calcium, adsorption

## Abstract

Cadmium (Cd), as a type of heavy metal, can increase the incidence of many diseases, even in low concentrations. In this study, tobermorite was hydrothermally synthesized and then applied to adsorb Cd^2+^ from an aqueous solution. The physicochemical characteristics of the synthesized tobermorite were detected, and the results indicated that the well-crystallized tobermorite had a lot of mesopores and a large specific surface area of 140.92 m^2^/g. It acquired a pH self-adjustment ability via spontaneously releasing Ca^2+^ and OH^-^ into the aqueous solution. The effects of different factors on Cd^2+^ removal were investigated. For Cd^2+^, the removal efficiency could reach 99.71% and the maximum adsorption capacity was 39.18 mg/g using tobermorite. The adsorption data was best fitted with the pseudo-second-order kinetic and Langmuir isotherm models. In addition, there was no strict limit on the solution pH in Cd^2+^ adsorption because the tobermorite could adjust the solution pH to an alkaline atmosphere spontaneously. The efficient removal of Cd^2+^ using tobermorite was a result of surface complexation and ion exchange.

## 1. Introduction

In recent decades, water demand has increased steadily due to population growth and industrial development [1]. Many parts of the world are now facing a water crisis, which may also be exacerbated by mismanagement of water resources [2,3]. Heavy metals are common in the effluents of industrial activities [4]. Excess levels of heavy metals in water resources will be a severe threat to the ecosystem and human health because they are highly toxic, bioconcentrated and nonbiodegradable [5]. In 1984, the United Nations Environment Program put forward 12 kinds of dangerous chemicals with global significance, of which Cd was ranked first [6]. Therefore, a remediation solution that can efficiently and economically remove Cd is essential for recycling and reutilizing of wastewater, which is also key to achieving sustainable management of water resources.

Numerous technologies have been employed to remove Cd, including chemical precipitation, adsorption, ion exchange, solvent extraction, reverse osmosis, coagulation/flocculation, photocatalysis and electrochemical treatment [7,8,9]. Among them, due to the simplicity, low cost and diversity of adsorbents, adsorption is regarded as promising [10,11]. However, some adsorbents still retain many disadvantages, such as poor structure, low metal selectivity and low adsorption capacity [12]. Da’na pointed out that the existence of Ca^2+^ and Na^+^ ions in adsorbent structures could enhance metal selectivity for some adsorbents [13]. Furthermore, heavy-metal removal is generally affected by solution pH [14,15]. For instance, a study by Sun and Yi indicated that concentrations of heavy-metal ions decreased when the pH was increased during the water-washing process of incineration bottom ash [14]. Therefore, it is a challenge to develop a novel, functional adsorbent that can release favorable ions (e.g., Na^+^ and Ca^2+^) and adjust solution pH simultaneously to make removal of Cd more efficient.

Tobermorite (Ca_5_Si_6_O_16_(OH)_2_·4H_2_O) is a type of naturally existing calcium silicate hydrate (CSH) [16]. It is widely applied in building materials (e.g., silica bricks, aerated concrete) because of its layered structure, large specific surface area, low density and thermal conductivity [17]. However, tobermorite products with low strength are easily broken during the production, transportation and application processes, and its waste usually accounts for about 15% of the total product [18]. Therefore, it is urgent to find appropriate recycling methods for tobermorite waste. In addition, depending on the hydration level, the (002) crystal plane of tobermorite can have different interplanar spacings, i.e., 0.9, 1.1 and 1.4 nm; thus, there are three types of tobermorite [18]. Numerous studies have synthesized 1.1 nm tobermorite under different hydrothermal conditions and using various raw materials [19,20,21]. A pore canal containing some water and Ca^2+^ ions is one of the most noticeable structural features of tobermorite [22]. Thus, tobermorite has recently been used to adsorb phosphorus at a laboratory scale [23,24,25]. The OH^-^ released from tobermorite provides a desired alkaline environment, which can facilitate the combination of Ca^2+^ and PO_4_^3-^ to form hydroxyl calcium phosphate [25]. Considering its layered structure and ability to release Ca^2+^ and OH^-^, tobermorite has the potential to become a functional adsorbent that removes Cd^2+^ from wastewater efficiently and economically [1]. However, previous studies have commonly focused on synthesis and modification (mainly Al substitution) of tobermorite but rarely on its pH self-adjustment ability or Cd^2+^ removal mechanism.

In this study, tobermorite was hydrothermally synthesized and its physicochemical properties, including crystalline phase, morphological features, pore size distribution and pH-adjustment ability, were characterized. Then, the variables influencing removal efficiency were explored using batch adsorption experiments. Lastly, the adsorption process was modeled, and the mechanism of Cd^2+^ removal using tobermorite was investigated.

## 2. Materials and Methods

### 2.1. Synthesis of Tobermorite

Tobermorite was synthesized hydrothermally according to the method of Zhao et al. [26]. Smelting quartz powder, which was produced from zirconium oxychloride waste, was the silicon source. Quicklime with an effective CaO content of 93%was the calcium source. The molar masses of calcium (Ca) in quicklime and silicon (Si) in the smelting quartz powder were respectively calculated, and the Ca/Si ratio was set to 0.83. The powder and the quicklime were mixed and ground for 15 min with water (solid/water = 1/15). Then, the mixture was digested at 90 °C for 2 h in a microautoclave and further autoclaved for 24 h at 1.6 MPa and 205 °C. Finally, tobermorite was acquired after drying at 105 °C.

### 2.2. Static Leaching Experiments

In order to evaluate the pH self-adjustment ability of tobermorite, 10 g of tobermorite was added to a conical flask with 1 L of deionized water. After sealing, the flask was agitated at 25 °C and 120 rpm in a water bath oscillator (HZQ-F100, Huamei, Jiangsu, China). The supernatant was collected at predetermined intervals (0.5, 1, 2, 4, 6, 8, 10 h) via filtering through a 0.45 μm microfiltration membrane. Ca^2+^ concentration was detected with EDTA titration. All reagents used were of analytical grade.

### 2.3. Batch Adsorption Experiments

A total of 1.6309 g of cadmium chloride (CdCl_2_) was dissolved in 1 L of deionized water to prepare Cd^2+^ stock solution (1000 mg/L). Different desired concentrations were obtained via diluting the stock solution using deionized water. Different parameters influencing the adsorption performance of the tobermorite were comprehensively investigated through a series of batch adsorption experiments.

In total, 0.5 g of tobermorite and 50 mL of Cd^2+^ solution with an initial concentration of 250 mg/L were added to a 100 mL polyvinyl chloride centrifugal tube. After sealing, the tube was agitated at 25 °C and 120 rpm in a water bath oscillator. For kinetics experiments, sampling time was predetermined at 0.5, 1, 2, 4, 6, 8, 10, 12 and 14 h. Then, a sampling time of 10 h was fixed for the analysis of other adsorption factors. Isotherm experiments were conducted at different initial Cd^2+^ concentrations of 50, 100, 150, 250, 400 and 500 mg/L. For different initial solution pH, the desired values (2.5, 4.5, 6.5, 8.5) were regulated using 1 M NaOH and HCl solutions. Tobermorite amounts of 2.5, 5, 10 and 15 g/L were used in the dosage experiment. Specifically, except for in the adsorption experiments at different initial Cd^2+^ concentrations, the Cd^2+^ concentration used was maintained at 250 mg/L with an initial solution pH of 6.6. Likewise, with the exception of the adsorption experiments at different initial solution pH, the solution pH was not additionally adjusted and kept at 6.6 in order to avoid metal hydroxide precipitation.

The supernatant was collected via filtering through a 0.45 μm microfiltration membrane. The Cd^2+^ concentration in the filtrates was analyzed with an atomic absorption spectrophotometer (AAS, Model TAS-990 F, Persee, Beijing, China). Results were obtained after three repetitions. Equations (1) and (2) are defined for removal efficiency (R, %) and adsorption capacity (Q_t_, mg/g), respectively:

(1) $$R=(C_{0}\;-\;C_{t})/C_{0}\;\times \;100\%$$

(2) $$Q_{t}=(C_{0}\;-\;C_{t})/M\;\times \;V\;$$

where, C_0_ (mg/L) is the initial Cd^2+^ concentration, C_t_ (mg/L) is the Cd^2+^ concentration at time t, V (L) is the solution volume and M (g) is the mass of tobermorite used.

### 2.4. Desorption Experiments

The adsorbed Cd^2+^ on tobermorite was desorbed with 0.1 M NaOH solution under 120 rpm and shaken for 1 h at 25 °C. Then, the tobermorite was washed with deionized water three times and used in the next adsorption cycle after drying. The adsorption–desorption cycle was conducted three times to investigate the reusability performance of tobermorite. In all of the adsorption experiments described here, the initial Cd^2+^ concentration was 250 mg/L, the contact time was 10 h and the adsorbent dosage was 0.5 g/50 mL.

### 2.5. Characterizations

The crystalline phase was analyzed with X-ray diffraction (XRD, D8 Advance, Bruker, Karlsruhe, Germany). Fourier transform infrared spectra (FTIR, Tensor 27, Bruker, Heidelberg, Germany) were obtained between 400 and 4000 cm^−1^ via adding samples in KBr pellets to observe characteristic function groups. The characteristics of pore structure were determined with a porosimeter (V-Sorb 28009, Gold APP, Beijing, China). A scanning electron microscope with energy dispersive spectroscopy (SEM-EDS, Gemini SEM 300, Zeiss, Jena, Germany) was applied to investigate morphological features and chemical composition. The zeta potential was obtained with a zeta potential meter (NanoBrook 90 Plus, Brookhaven, Suffolk County, NY, USA).

## 3. Results and Discussion

### 3.1. Characterizations of Tobermorite

**Figure 1:**
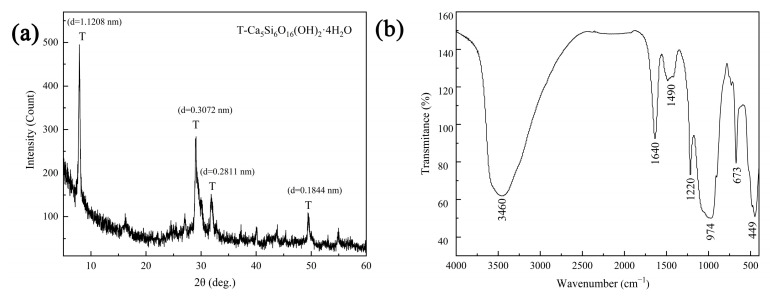
XRD pattern (**a**) and FTIR spectrum (**b**) of tobermorite.

The XRD pattern in Figure 1a indicates that tobermorite with good crystallinity was synthesized via the hydrothermal method. At 2θ angles of 7.9°, 29.0°, 31.8° and 49.4°, diffraction peaks for the lattice planes of (002), (110), (107) and (217) were found in orthorhombic tobermorite (PDF #83-1520, d_1_ = 1.1389 nm, d_2_ = 0.3082 nm), which could be identified as 1.1 nm tobermorite [19]. The FTIR spectrum of tobermorite is exhibited in Figure 1b. The broad band at 3460 cm^−1^ was attributed to O-H stretching vibration, which was generated from the adsorbed H_2_O molecules and the surface hydroxyls [27]. The band at 1640 cm^−1^ was assigned to O-H bending vibration. The small band at 1490 cm^−1^ was caused by H_2_O molecules in the interlayers of tobermorite [28]. The broad band near 974 cm^−1^ was attributed to Si-O symmetric stretching vibration and Si-O-Si asymmetric vibration in the silica tetrahedron. The band at 449 cm^−1^ was due to Si-O bending vibration in the silica tetrahedron [19]. Therefore, consistently with the above XRD analysis, tobermorite with a high degree of crystallization was synthesized successfully in this study.

**Figure 2:**
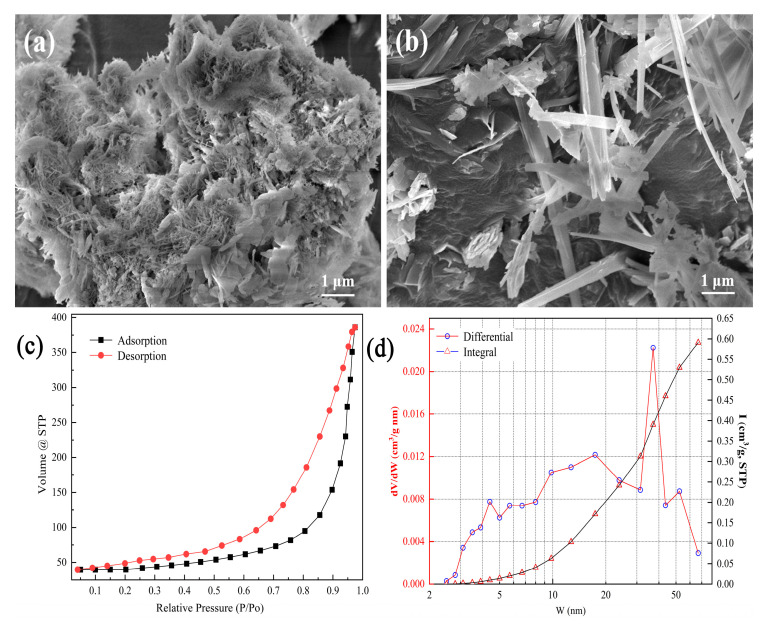
(**a**,**b**) SEM images, (**c**) nitrogen adsorption–desorption isotherms and (**d**) pore size distribution of tobermorite.

The surface morphology of tobermorite, presented in Figure 2a,b, mainly consists of micron-grade rod- and sheet-like structures. The two SEM images show different crystal growth densities in the tobermorite. In Figure 2a, most of the tobermorite crystals are small and rod-like due to limited growth space. When there was enough growth space, tobermorite crystals could grow into a sheet-like structure, as shown in Figure 2b. Regardless, numerous void spaces were still found in the tobermorite, which was beneficial for Cd^2+^ adsorption [26]. The adsorption–desorption isotherms of tobermorite in Figure 2c conform to the typical features of a type IV isotherm with an H3 hysteresis loop, suggesting that large and narrow slit-like pores were formed between the tobermorite crystals [29]. The pore distribution curve in Figure 2d indicated that mesopores of about 30–45 nm were predominant in the tobermorite, which could in turn explain the relatively large hysteresis loop shown in Figure 2c. In addition, the tobermorite’s surface area was 140.92 m^2^/g (BET method), its total pore volume was 0.60 cm^3^/g and its average pore width was 16.95 nm. Generally, porous materials with large surface areas can provide more adsorption and ion exchange sites, and thus, tobermorite should be a promising adsorbent [30].

The hydration of the tobermorite is described in Equation (3), showing that Ca^2+^ and OH^−^ ions could be released into deionized water simultaneously [31]:

Ca_5_Si_6_O_16_(OH)_2_·4H_2_O → Ca^2+^ + OH^−^ + CSH (3)

**Figure 3:**
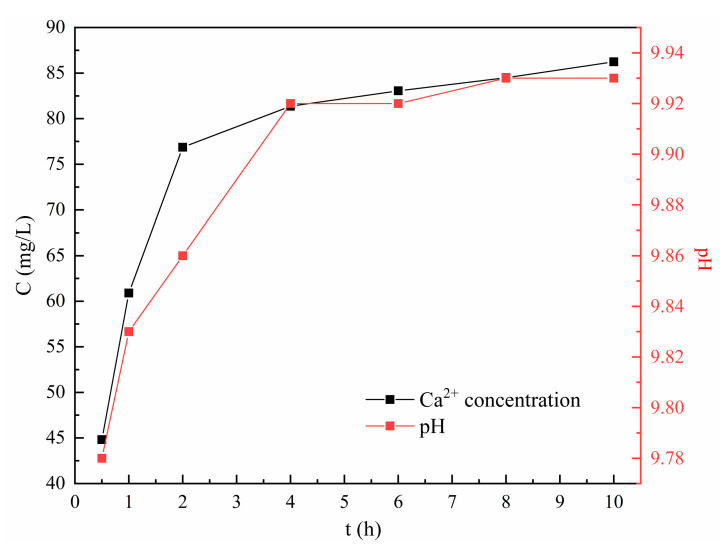
Releasing ability of tobermorite.

Thus, the changes in Ca^2+^ concentration and solution pH during the static leaching experiments are described in Figure 3. An increase in Ca^2+^ concentration was prominent within the first 2 h and then slowed down significantly. After 10 h, 86.24 mg/L of Ca^2+^ was detected, accounting for 3.15% of the total Ca mass in the tobermorite used. Meanwhile, the solution pH was promptly increased to 9.8 within 0.5 h, then remained at about 9.9 after 4 h, indicating that an alkaline environment in the solution was autonomously created from the tobermorite. Thus, the tobermorite was considered to have a pH self-adjustment ability. In the structure of tobermorite, the layers consisting of SiO_4_ tetrahedral chain–Ca polyhedral layer–SiO_4_ tetrahedral chain laminates along the c-axis formed the interlayer space through shared SiO_4_ tetrahedrons [32]. Ca^2+^ ions in interlayer space are inclined to be dissolved or substituted with some suitable heavy metals [22]. Wang et al. reported that the decalcification of CSH in weak acidic environments did not affect its morphology [33]. Considering that the structures of CSH and tobermorite are similar, it was reasonable to infer that the small amount of Ca^2+^ ions dissolved in this study would not affect the morphology of tobermorite.

### 3.2. Effects of Variables on Cd^2+^ Removal Using Tobermorite

#### 3.2.1. Effect of Adsorbent Dosage

**Figure 4:**
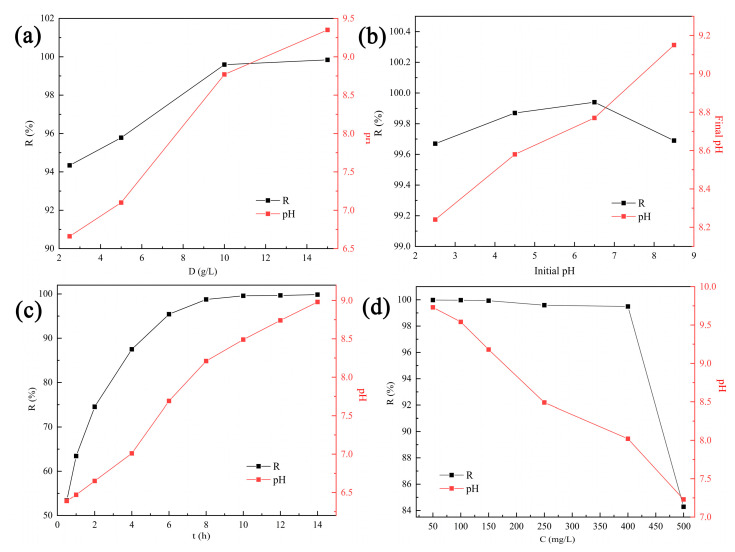
Removal efficiency of Cd^2+^ and solution pH at different (**a**) dosages, (**b**) initial solution pH values, (**c**) contact times and (**d**) initial Cd^2+^ concentrations.

As illustrated in Figure 4a, when the tobermorite dosage increased from 2.5 to 10 g/L, the removal efficiency of Cd^2+^ was increased from 94.34% to 99.71%. Increased tobermorite dosages could provide more active sites to promote Cd^2+^ removal [34]. However, no significant improvement was observed after the dosage was increased from 10 to 15 g/L. Hence, considering economy and high efficiency, 10 g/L was selected as the optimum dosage and generalized in the following experiments.

#### 3.2.2. Effect of Initial Solution pH

**Figure 5:**
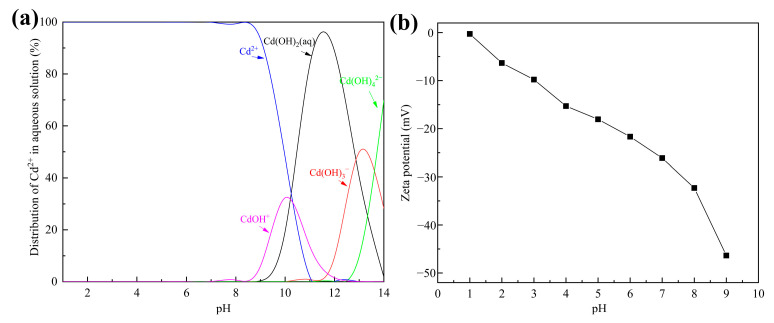
(**a**) The distribution of Cd^2+^ in aqueous solution simulated with Visual MINTEQ 3.1; (**b**) zeta potential of tobermorite at different initial solution pH values.

Solution pH can affect the states of adsorbents and ions simultaneously, so it is an important factor in removal of heavy metals from wastewater [35]. Distribution of Cd^2+^ substances under different pH values was simulated with Visual MINTEQ 3.1 and is shown in Figure 5a. Clearly, Cd^2+^ was the major substance when the pH was below 8.5. Considering that Cd^2+^ ions are inclined to form precipitation at higher pH values, this study was carried out in an initial pH range of 2.5–8.5.

The removal efficiency of Cd^2+^ using tobermorite and the final solution pH at different initial pH values are depicted in Figure 4b. Interestingly, the tobermorite exhibited a considerably high removal efficiency of 99.67–99.94% for Cd^2+^ in the whole pH range investigated. Even in an acid solution with a relatively low initial pH of 2.5, the removal efficiency still reached up to 99.67%. The obtained result was inconsistent with most of the previous research, which had indicated that initial solution pH significantly affected removal efficiency of heavy metals [36]. 

The zeta potential of tobermorite is displayed in Figure 5b. In the investigated pH range of 2.5–8.5, the surface charge of the tobermorite was always negative and decreased from about −9 to −40 mV. Electrostatic repulsion to positively charged Cd^2+^ was not produced because there was no positive charge on the surface of the tobermorite, which ensured high removal efficiency of Cd^2+^. Generally, the higher negative charge on the surface of tobermorite could significantly enhance electrostatic attraction to Cd^2+^, resulting in increased removal efficiency of Cd^2+^. In fact, the removal efficiency of Cd^2+^ increased by only 0.27% from initial pH values of 2.5 to 6.5. Compared with the corresponding high removal efficiency, this slight increase was negligible. The high removal efficiency of Cd^2+^ at low initial pH might be attributed to the pH self-adjustment ability of tobermorite. As shown in Figure 4b, the final solution pH was adjusted to a range of 8.2–9.2 using tobermorite. OH^−^ ions were released from the tobermorite through the hydration process, as described in Equation (3), and captured H^+^ continuously, so a relatively stable alkaline environment was ensured. The ultimate result was that the tobermorite could adsorb Cd^2+^ efficiently over a wide pH range. In a practical application, this would mean that pH adjustment reagents are unnecessary when tobermorite is used, which could simplify the operation and reduce costs.

#### 3.2.3. Effects of Contact Time and Adsorption Kinetics

**Figure 6:**
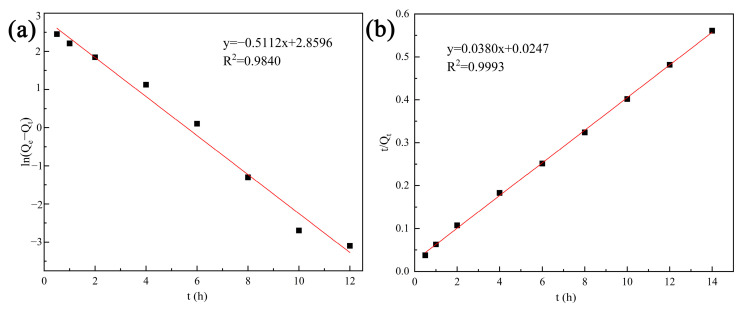
Kinetic models for Cd^2+^ removal using tobermorite: (**a**) the PFO model and (**b**) the PSO model.

The effect of contact time on Cd^2+^ removal using tobermorite is shown in Figure 4c. Removal efficiency was notoriously increased with contact time due to the presence of sufficient active sites on the surface. Then, the adsorption equilibrium was reached at 8 h with a removal efficiency of nearly 99.62%. At the same time, the solution pH increased persistently, indicating that the tobermorite could release OH^−^ independently to regulate the pH. The pseudofirst-order (PFO) and pseudosecond-order (PSO) kinetic models, as described in Equations (4) and (5), were used to fit the kinetic data [37]. The linear plots and obtained parameters are presented in Figure 6 and Table 1, respectively. Compared with the PFO model, the correlation coefficient (R^2^ = 0.9993) of the PSO model was much higher. In addition, the theoretical value of Q_e_ (26.32 mg/L) calculated from the PSO model was closer to the experimental observation (24.97 mg/L). Therefore, Cd^2+^ removal using tobermorite is better described with the PSO model, and the process tended to be chemical adsorption [38];

ln(Q_e_ − Q_t_) = lnQ_e_ − K_1_t (4)

t/Q_t_ = 1/(K_2_Q_e_^2^) + t/Q_e_ (5)

where t (h) is the contact time; Q_e_ (mg/g) is the adsorption capacity at equilibrium; and k_1_ (1/h) and k_1_ (1/h) are the rate constants of the PFO and PSO models, respectively. 

#### 3.2.4. Effects of Initial Cd^2+^ Concentration and Adsorption Isotherms

The initial concentration of contaminants also exerts a critical influence during adsorption; hence, solutions with different initial Cd^2+^ concentrations were investigated, as exhibited in Figure 4d. The removal efficiency remained at a high level of above 99.49% within an initial concentration range of 50–400 mg/L. When the Cd^2+^ concentration was further increased to 500 mg/L, the removal efficiency decreased to 84.28%. However, the adsorption capacity persistently increased with the increasing Cd^2+^ concentration and reached a maximum value of 42.14 mg/g at the end. In addition, the solution pH after adsorption decreased from 9.7 to 7.2 with the increase in initial Cd^2+^ concentration. It can be inferred that the release of OH^-^ from tobermorite might be inhibited in solutions with high Cd^2+^ concentrations. Isotherm data obtained in this study were analyzed using the Langmuir and Freundlich isotherm models, which are described in Equations (6) and (7), respectively [37]:

C_e_/Q_e_ = 1/(Q_m_K_L_) + C_e_/Q_m_ (6)

lnQ_e_ = lnK_F_ + nlnC_e_ (7)

where C_e_ (mg/L) is the equilibrium concentration; Q_e_ (mg/g) and Q_m_ (mg/g) are the equilibrium and maximum adsorption capacities; K_L_ (L/mg) and K_F_ ((mg/g)/(mg/L)^1/n^) are the adsorption constants of the Langmuir and Freundlich models, respectively; and n is the empirical constant related to adsorption strength.

**Figure 7:**
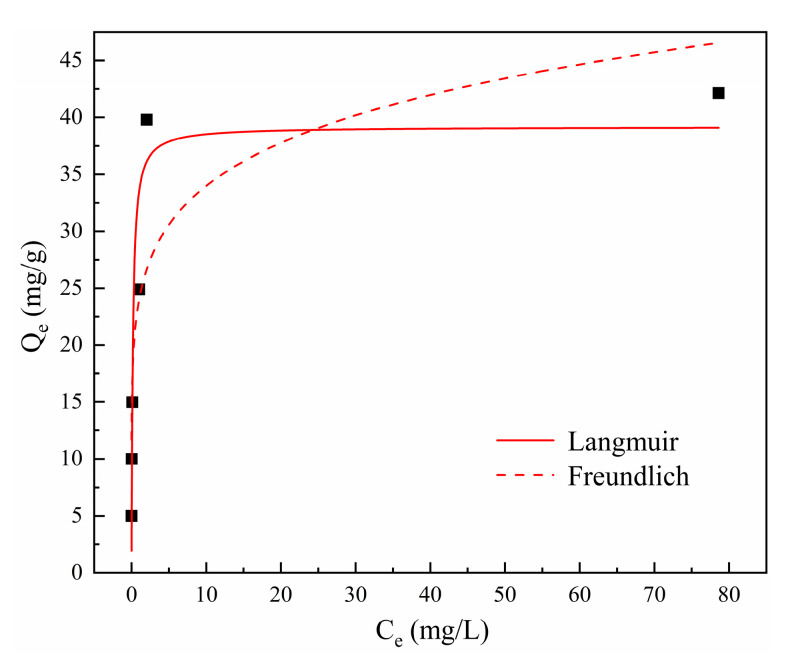
Isotherm models for Cd^2+^ removal using tobermorite.

The nonlinear plots for both isotherm models are presented in Figure 7, and the calculated parameters are in Table 2. It was clear that Cd^2+^ removal using tobermorite was well described with the Langmuir model (R^2^ = 0.8983), which suggested that Cd^2+^ removal was a process of monolayer chemical adsorption that probably occurred through surface complexation with tobermorite [2,39]. Different studies also showed that Cd^2+^ removal with a range of other adsorbents was fitted better with the Langmuir model than with the Freundlich model [35,40,41]. In addition, Q_m_, as the maximum adsorption capacity calculated from the Langmuir model, could be used as the theoretical saturated adsorption capacity. The Q_m_ for Cd^2+^ removal using tobermorite was 39.18 mg/g, which was relatively consistent with the experimental result of 42.14 mg/g. Table 3 gives a comparison of Q_m_ values for Cd^2+^ adsorption for various relevant adsorbents. It could be noticed that tobermorite in this study outperformed other relevant adsorbents, since it had the highest Q_m_. Among them, the adsorption capacity of thermally treated limestone for Cd^2+^ was close to that of tobermorite. However, this method generally had the problem of high pH and Ca^2+^ concentration in the treated effluent, so it was usually used with other reagents or techniques in practice. In addition, tobermorite waste, such as broken silica bricks and aerated concrete, is considered to be a source of tobermorite for practical wastewater treatment applications. As a consequence, tobermorite could be considered as a more pre-eminent and economical adsorbent for Cd^2+^ in wastewater.

### 3.3. Mechanism of Cd^2+^ Removal Using Tobermorite

**Figure 8:**
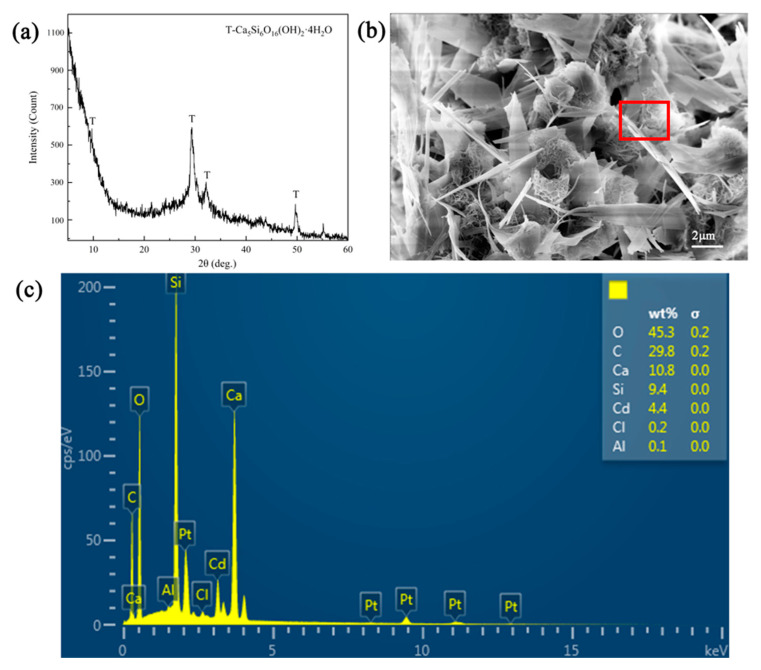
(**a**) XRD pattern, (**b**) SEM and (**c**) EDS (the detection area was in the red box of (**b**)) images of tobermorite after Cd^2+^ adsorption.

In order to explore the adsorption mechanism of Cd^2+^ removal using tobermorite, the XRD pattern of tobermorite after adsorption was detected, as shown in Figure 8a. The diffraction peaks associated with tobermorite remained, while the intensity of some peaks, especially the peak at 2θ = 7.9°, was reduced. It could be inferred that the ion exchange of Cd^2+^ and Ca^2+^ indeed occurred and affected the crystal structure of the tobermorite to some extent. Additionally, the new diffuse peak in the range of 2θ = 5–15° indicated that a certain amount of amorphous phase was generated after adsorption. Some studies reported that CSH would generate during the hydration process of tobermorite, as described in Equation (3) [31,46]. Zou et al. also revealed that the ion exchange between heavy metals and Ca^2+^ in tobermorite could lead to crystallinity damage, i.e., amorphization [47]. 

Compared with the morphology of tobermorite before adsorption in Figure 2a,b, tobermorite after Cd^2+^ adsorption in Figure 8b still consisted of micron-sized rod- and sheet-like particles ascribing to its high stability. In addition, some small flocculent and clumpy structures appeared after adsorption; this might be associated with the amorphous phase. The EDS analysis in Figure 8c clearly shows that 4.40 wt% of Cd was detected on the surface of the tobermorite, demonstrating that Cd^2+^ was adsorbed.

**Figure 9:**
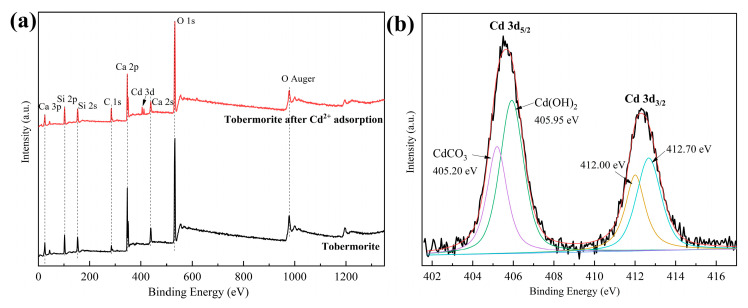
(**a**) XPS survey scans of tobermorite before and after Cd^2+^ adsorption; (**b**) high-resolution XPS spectrum of Cd 3d.

XPS was employed to further compare tobermorite before and after adsorbing Cd^2+^. From the survey spectra in Figure 9a, signals of Ca, Si and O, which belonged to tobermorite, were observed before adsorption, and a new signal of elemental Cd appeared after adsorption, further validating the hypothesis of effective Cd^2+^ adsorption into tobermorite. Additionally, compared with tobermorite before adsorption, the peak intensities of Ca after adsorption decreased slightly, indicating that Ca participated in Cd^2+^ adsorption. The decrease was mainly due to the leaching of Ca^2+^ from the tobermorite through ion exchange [48,49]. The high-resolution XPS spectrum shown in Figure 9b is for Cd 3d. There were two broad and asymmetric peaks at 405.50 and 412.35 eV, which were attributable to Cd 3d_5/2_ and Cd 3d_3/2_, respectively. The subpeaks deconvoluted from Cd 3d_5/2_ were at 405.20 and 405.95 eV, and those from Cd 3d_3/2_ were at 412.00 and 412.70 eV, respectively. The two sets of subpeaks had a peak separation of ~6.8 eV, which could be attributed to CdCO_3_ and Cd(OH)_2_, respectively [50]. Therefore, it could be inferred that the monolayer of complex surface substances consisted of CdCO_3_ and Cd(OH)_2_.

**Figure 10:**
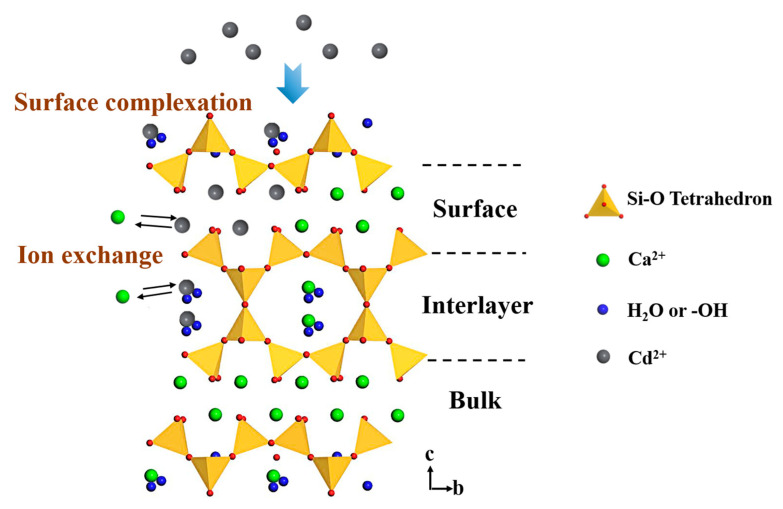
Mechanism of Cd^2+^ removal using tobermorite.

Figure 10 presents the possible mechanism of Cd^2+^ removal using tobermorite. The tobermorite synthesized in this study had a high surface area and could release OH^−^ spontaneously, which could have contributed to Cd^2+^ adsorption through surface complexation in the forms of Cd(OH)_2_ and CdCO_3_ first. Afterwards, when the Cd^2+^ concentration on the surface of the tobermorite reached a certain extent, ion exchange between Cd^2+^ and Ca^2+^ would occur on the surface. In addition, Cd^2+^ could diffuse into the tobermorite through the mesopores and exchange with Ca^2+^ in the interlayer space, which would be much easier than exchanging with Ca^2+^ in the bulk space of the tobermorite.

### 3.4. Reusability Performance of Tobermorite

**Figure 11:**
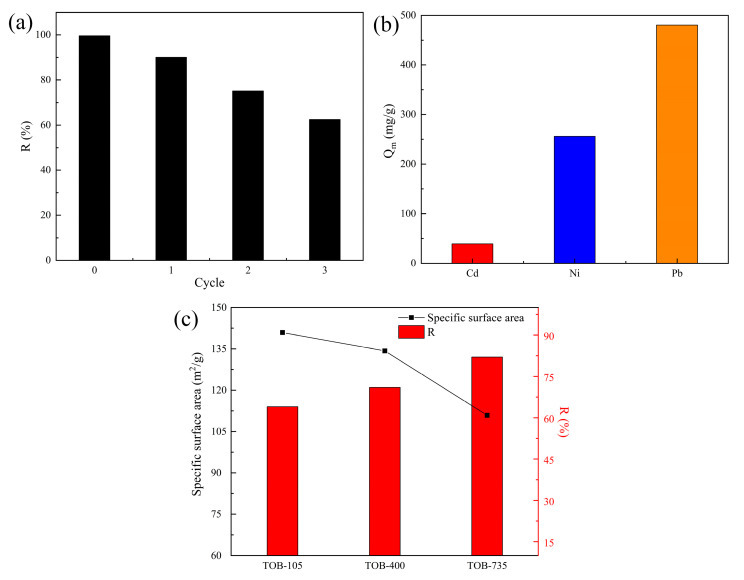
(**a**) Reusability of tobermorite for Cd^2+^ adsorption. (**b**) Q_m_ values of tobermorite for Cd^2+^, Ni^2+^ and Pb^2+^. (**c**) Specific surface areas and removal efficiency of Ni^2+^ with different modified tobermorites.

Tobermorite, as a kind of CSH, has the tendency of being dissolved in strongly acidic environments, so structure destruction may be caused by continuous use of acidic eluents. In this study, the reusability and stability of tobermorite were investigated through conducting consecutive Cd^2+^ adsorption–desorption experiments using 0.1 M NaOH as an eluent. As displayed in Figure 11a, the removal efficiency of Cd^2+^ using tobermorite decreased from 99.69% to 62.47% after three adsorption–resolution cycles. This result might be due to the strong interaction between Cd^2+^ and tobermorite and the gradual consumption of the active sites and Ca^2+^ available. From an application point of view, the reuse cycle of two to three times was more suitable for tobermorite in Cd^2+^ adsorption, and performing more cycles was of little economic significance due to poor removal efficiency and adsorption capacity. Nonetheless, after the last cycle, Cd^2+^ could be recovered with desorption and the residual tobermorite could be recycled as a raw material to prepare new, environmentally friendly materials, such as sintered silicate ceramsites and bricks, according to our previous studies [51,52]. This strategy could recycle Ca and Si resources and simultaneously immobilize the residual Cd in tobermorite via sintering at high temperatures.

### 3.5. Ni^2+^ and Pb^2+^ Removal Using Tobermorite

In order to investigate the adsorption universality of tobermorite for heavy metals, two other typical divalent heavy-metal ions, Ni^2+^ and Pb^2+^, were selected to conduct batch adsorption experiments at different initial Ni^2+^/Pb^2+^ concentrations. The isotherm data obtained therein was also well fitted with the Langmuir model, and the Q_m_ values calculated for Ni^2+^ and Pb^2+^ were 256.31 and 480.02 mg/g, respectively, as shown in Figure 11b. It can be concluded that tobermorite is an efficient and universal adsorbent for most heavy-metal ions. Moreover, the Q_m_ values for Ni^2+^ and Pb^2+^ were clearly higher than that for Cd^2+^, which might be attributed to the different removal mechanisms and affinity of tobermorite for different heavy-metal ions. Further investigation and explanation of this phenomenon will be the focus of our future study.

Generally, a higher specific surface area of an adsorbent means more porosity and more abundant active sites, which may contribute to a higher adsorption capacity for contaminants [53]. However, in some specific cases, specific surface area and adsorption capacity are not necessarily proportional to each other, so more factors need to be taken into account. For example, Guo et al. found that Al substitution would decrease the specific surface area of tobermorite from 198.83 m^2^/g to 80.48 m^2^/g through refining pore structure and particle size; at the same time, it was found that the adsorption capacities of Pb^2+^ and Cu^2+^ would decrease, but the adsorption capacity of Cr^6+^ would increase [54]. Wang et al. synthesized tobermorite hydrothermally from fly ash at different reacting temperatures (180–260 °C) to remove Pb^2+^. These results indicated that tobermorite could obtain its highest specific surface area of 31.04 m^2^/g at 200 °C, while the optimal adsorption capacity for Pb^2+^ was reached at 180 °C [21]. In this study, the possible relationship between specific surface area and adsorption capacity for heavy metals was also explored using different tobermorites. The tobermorite prepared (dried at 105 °C) was further calcined at 400 and 735 °C; these samples were labeled as TOB-105, TOB-400 and TOB-735, respectively. As shown in Figure 11c, the specific surface areas of the three tobermorites were 140.92, 134.25 and 110.89 m^2^/g, respectively. During the calcination process, the 1.1 nm tobermorite was gradually transformed into 0.9 nm tobermorite through dehydration and dehydroxylation [53], resulting in a decrease in the number of porous channels and specific surface area. The adsorption experiments were carried out using the three tobermorites at an initial Ni^2+^ concentration of 400 mg/L, an adsorbent dosage of 50 mg/50 mL and a contact time of 6 h. The removal efficiency of Ni^2+^ for each sample was 64.11, 71.23 and 82.05% respectively, as displayed in Figure 11c. This could be attributed to the activation of Ca^2+^ in tobermorite at high temperatures [54], which increases the capacity of ion exchange between Ca^2+^ and Ni^2+^ during adsorption. Considering that the removal mechanism of Ni^2+^ (surface complexation–ion exchange) was similar to that of Cd^2+^ using tobermorite, an improvement in the adsorption capacity for Cd^2+^ could be achieved through activating Ca^2+^ in tobermorite in our future study. It could also be inferred that the adsorption performance of some adsorbents with considerably high specific surface areas could be optimized through enhancement of some specific functions rather than through continuous improvement of specific surface area.

## 4. Conclusions

In this study, with smelting quartz powder (waste material) and quicklime used as raw materials, tobermorite with pH self-adjustment ability was successfully synthesized, and its potential application for Cd^2+^ removal was investigated. It was found that the well-crystallized tobermorite consisted of micron-sized rod- and sheet-like structures, resulting in formation of mesopores and a large specific surface area of 140.92 m^2^/g. An alkaline environment in a solution could be created from tobermorite autonomously due to its hydration. Batch adsorption experiments proved that tobermorite had a high removal efficiency of 99.71% for Cd^2+^, and its kinetic data was best fitted with the PSO model. The Langmuir model provided the best description for its isotherm data, and the Q_m_ was 39.18 mg/g. In addition, the removal efficiency of Cd^2+^ was kept at a high level, above 99.6%, in an initial pH range of 2.5–8.5. The removal mechanism of Cd^2+^ involved surface complexation with OH^-^ released from tobermorite as well as ion exchange with Ca^2+^ from the surface and the interlayer space of the tobermorite. In addition, an adsorption–desorption cycle of two to three times was found to be more suitable for tobermorite in Cd^2+^ adsorption, whereas tobermorite after final desorption could be recycled as a raw material to prepare new, environmentally friendly materials, such as sintered silicate ceramsites and bricks, making it attractive from a sustainability point of view. The tobermorite also had high Q_m_ values of 256.31 and 480.02 mg/g for Ni^2+^ and Pb^2+^, respectively, indicating that it had a good adsorption universality for additional heavy metals. Consequently, this study not only indicated that tobermorite is an effective and low-cost adsorbent for Cd^2+^ but also provided a feasible way to recycle torbermorite waste.

## Figures and Tables

**Table 1:** Kinetic model parameters for Cd^2+^ removal using tobermorite.

Q_e,exp_	PFO Model			PSO Model		
24.97	K_1_	Q_e_	R^2^	K_2_	Q_e_	R^2^
	0.51	17.45	0.9840	0.06	26.32	0.9993

**Table 2:** Isotherm model parameters for Cd^2+^ removal using tobermorite.

Q_m,exp_	Langmuir Isotherm Model			Freundlich Isotherm Model		
	Q_m_	K_L_	R^2^	n	K_F_	R^2^
42.14	39.18	5.82	0.8983	0.15	23.93	0.7873

**Table 3:** Comparison of Q_m_ for Cd^2+^ using relevant adsorbents.

Adsorbent	Q_m_ (mg/g)	Reference
Quartz sand	30.77	[42]
Goethite-coated sand	0.15–0.2	[43]
Thermally treated limestone	37.19	[44]
Al-substituted tobermorite	2.92	[45]
Tobermorite	39.18	This study

## Data Availability

Not applicable.

## References

[1] Cardinale A.M., Carbone C., Fortunato M., Fabiano B., Reverberi A.P. (2022). ZnAl-SO_4_ layered double hydroxide and allophane for Cr(VI), Cu(II) and Fe(III) adsorption in wastewater: Structure comparison and synergistic effects. Materials.

[2] Takaya Y., Kadokura M., Kato T., Tokoro C. (2021). Removal mechanisms of arsenite by coprecipitation with ferrihydrite. J. Environ. Chem. Eng..

[3] Sirohi R., Joun J., Lee J.Y., Yu B.S., Sim S.J. (2022). Waste mitigation and resource recovery from food industry wastewater employing microalgae-bacterial consortium. Bioresour. Technol..

[4] Tejada-Tovar C., Villabona-Ortiz A., González-Delgado Á. (2022). Adsorption study of continuous heavy metal ions (Pb^2+^, Cd^2+^, Ni^2+^) removal using cocoa (*Theobroma cacao* L.) pod husks. Materials.

[5] Islam M.A., Morton D.W., Johnson B.B., Mainali B., Angove M.J. (2018). Manganese oxides and their application to metal ion and contaminant removal from wastewater. J. Water Process Eng..

[6] Du B.Y., Zhou J., Lu B.X., Zhang C., Li D.M., Zhou J., Jiao S.J., Zhao K.Q., Zhang H.H. (2020). Environmental and human health risks from cadmium exposure near an active lead-zinc mine and a copper smelter. Sci. Total Environ..

[7] Suzuki K., Kato T., Fuchida S., Tokoro C. (2020). Removal mechanisms of cadmium by δ-MnO_2_ in adsorption and coprecipitation processes at pH 6. Chem. Geol..

[8] Fuchida S., Tajima S., Tokoro C. (2021). Surface complexation modeling of Cd on Mn(III) oxyhydroxide (γ-MnOOH) for neutralizing model of acid mine drainage. Resour. Process..

[9] Qiu B.B., Tao X.D., Wang H., Li W.K., Ding X., Chu H.Q. (2021). Biochar as a low-cost adsorbent for aqueous heavy metal removal: A review. J. Anal. Appl. Pyrolysis.

[10] Lu Z.X., Li X.Z., Qi X.J. (2021). Cobalt-loaded resin can effectively remove arsenic in wastewater. Environ. Technol. Innov..

[11] Muñoz S.V., Martínez M.S., Torres M.G., Alcalá S.P., Quintanilla F., Rodríguez-Canto A., Rodríguez J.R. (2014). Adsorption and removal of cadmium ions from simulated wastewater using commercial hydrophilic and hydrophobic silica nanoparticles: A comparison with sol-gel particles. Water Air Soil Pollut..

[12] Farooq U., Kozinski J.A., Khan M.A., Athar M. (2010). Biosorption of heavy metal ions using wheat based biosorbents-a review of the recent literature. Bioresour. Technol..

[13] Da’na E. (2017). Adsorption of Heavy Metals on Functionalized-Mesoporous Silica: A Review. Microporous Mesoporous Mat..

[14] Sun X.L., Yi Y.L. (2020). pH evolution during water washing of incineration bottom ash and its effect on removal of heavy metals. Waste Manag..

[15] Jiang S.S., Huang L.B., Nguyen T.A.H., Ok Y.S., Rudolph V., Yang H., Zhang D.K. (2016). Copper and zinc adsorption by softwood and hardwood biochars under elevated sulphate-induced salinity and acidic pH conditions. Chemosphere.

[16] Komarneni S., Komarneni J.S., Newalkar B., Stout S. (2002). Microwave-hydrothermal synthesis of Al-substituted tobermorite from zeolites. Mater. Res. Bull..

[17] Liao L.N., Zhang P. (2018). Preparation and characterization of polyaluminum titanium silicate and its performance in the treatment of low-turbidity water. Processes.

[18] Mitra N., Sarkar P.K., Prasad D. (2019). Intermolecular dynamics of ultraconfined interlayer water in tobermorite: Influence on mechanical performance. Phys. Chem. Chem. Phys..

[19] Liao W., Li W.Q., Fang Z.G., Lu C.H., Xu Z.Z. (2019). Effect of different aluminum substitution rates on the structure of tobermorite. Materials.

[20] Siauciunas R., Smalakys G., Eisinas A., Prichockiene E. (2022). Synthesis of high crystallinity 1.13 nm tobermorite and xonotlite from natural rocks, their properties and application for heat-resistant products. Materials.

[21] Wang Z.H., Xu L.H., Wu D.S., Zheng S.L. (2022). Hydrothermal synthesis of mesoporous tobermorite from fly ash with enhanced removal performance towards Pb^2+^ from wastewater. Colloid Surf. A-Physicochem. Eng. Asp..

[22] Monasterio M., Gaitero J.J., Manzano H., Dolado J.S., Cerveny S. (2015). Effect of chemical environment on the dynamics of water confined in calcium silicate minerals: Natural and synthetic tobermorite. Langmuir.

[23] Moriyama K., Kojima T., Minawa Y., Matsumoto S., Nakamachi K. (2001). Development of artificial seed crystal for crystallization of calcium phosphate. Environ. Technol..

[24] Jin A.F., He J.T., Zheng H., Huang G.X. (2009). Experimental study on treating low phosphorus wastewater by combination of tobermorite and calcium fluoride. Environ. Sci. Technol..

[25] Tian W., Shui A., Ke S.J., Huang L.L., Xi X., He C., Chen W.W., Du B. (2019). Low-temperature preparation of humidity self-regulating porous ceramics with high strength performance. Mater. Lett..

[26] Zhao Q.Y., Li T., Cui C., Wang Z.Z., Ding X.F., Zhang S.H. (2020). Preparation of porous silica powder via selective acid leaching of calcined tobermorite. Powder Technol..

[27] Mahmoud H.R., El-Molla S.A., Saif M. (2013). Improvement of physicochemical properties of Fe_2_O_3_/MgO nanomaterials by hydrothermal treatment for dye removal from industrial wastewater. Powder Technol..

[28] Li R.C., Zheng S.L., Sun Z.M., Li C.Q. (2021). Research on synthesis of flaky tobermorite from diatomite and its mechanism. Inorg. Chem. Ind..

[29] Zheng X.G., Gou Y., Peng H., Mao Y.T., Wen J. (2021). Nonthermal plasma sulfurized CuInS_2_/S-doped MgO nanosheets for efficient solar-light photocatalytic degradation of tetracycline. Colloid Surf. A-Physicochem. Eng. Asp..

[30] Zhu X.H., Jia X.H. (2020). Removal of chromium (III) from monoammonium phosphate solutions by a porous adsorbent of fluor (calcium silicate) composites. J. Wuhan Univ. Technol.-Mat. Sci. Edit..

[31] Cao P.X., Li G.H., Luo J., Rao M.J., Jiang H., Peng Z.W., Jiang T. (2020). Alkali-reinforced hydrothermal synthesis of lathy tobermorite fibers using mixture of coal fly ash and lime. Constr. Build. Mater..

[32] Kamei S., Ihara T., Ouchi T., Uzawa M., Machinaga O. (2014). A novel synthesis of phosphorus-substituted tobermorite with calcium silicate hydrate. J. Ceram. Soc. Jpn..

[33] Wang X.B., Pan Z.H. (2017). Chemical changes and reaction mechanism of hardened cement paste-(NH_4_)_2_SO_4_-H_2_O-system. Constr. Build. Mater..

[34] Dai S.W., Wen Q., Huang F., Bao Y.Q., Xi X.D., Liao Z.P., Shi J., Ou C.J., Qin J. (2022). Preparation and application of MgO-loaded tobermorite to simultaneously remove nitrogen and phosphorus from wastewater. Chem. Eng. J..

[35] Shi Q.L., Zhang H., Shahab A., Zeng H.H., Zeng H.T., Bacha A.U.R., Nabi I., Siddique J., Ullah H. (2021). Efficient performance of magnesium oxide loaded biochar for the significant removal of Pb^2+^ and Cd^2+^ from aqueous solution. Ecotox. Environ. Safe..

[36] Ianăşi C., Picioruş M., Nicola R., Ciopec M., Negrea A., Nižňanský D., Len A., Almásy L., Putz A.M. (2019). Removal of cadmium from aqueous solutions using inorganic porous nanocomposites. Korean J. Chem. Eng..

[37] Ou C.J., Dai S.W., Li S.X., Xu J., Qin J. (2021). Adsorption performance and mechanism investigation of Mn2+ by facile synthesized ceramsites from lime mud and coal fly ash. Korean J. Chem. Eng..

[38] Bhanjana G., Dilbaghi N., Kim K.H., Kumar S. (2017). Carbon nanotubes as sorbent material for removal of cadmium. J. Mol. Liq..

[39] Tokoro C., Kato T. (2021). Arsenate removal by resin-supported ferric ions: Mechanism, modeling, and column study. Adv. Powder Technol..

[40] Javaheri F., Kheshti Z., Ghasemi S., Altaee A. (2019). Enhancement of Cd^2+^ removal from aqueous solution by multifunctional mesoporous silica: Equilibrium isotherms and kinetics study. Sep. Purif. Technol..

[41] Takdastan A., Samarbaf S., Tahmasebi Y., Alavi N., Babaei A.A. (2019). Alkali modified oak waste residues as a cost-effective adsorbent for enhanced removal of cadmium from water: Isotherm, kinetic, thermodynamic and artificial neural network modeling. J. Ind. Eng. Chem..

[42] Schütz T., Dolinská S., Hudec P., Mockovčiaková A., Znamenáčková I. (2016). Cadmium adsorption on manganese modified bentonite and bentonite-quartz sand blend. Int. J. Miner. Process..

[43] Lai C.H., Chen C.Y., Wei B.L., Yeh S.H. (2002). Cadmium adsorption on goethite-coated sand in the presence of humic acid. Water Res..

[44] Kang K., Gu B.W., Kim Y.K., Park S.J. (2016). Removal of Cu^2+^, Cd^2+^ from water using thermally treated lime stone. KSWST Jour. Wat. Treat..

[45] Coleman N.J., Brassington D.S., Raza A., Lee W.E. (2006). Calcium silicate sorbent from secondary waste ash: Heavy metals-removal from acidic solutions. Environ. Technol..

[46] Chen M.X., Lu L.C., Wang S.D., Zhao P.Q., Zhang W.L., Zhang S.X. (2017). Investigation on the formation of tobermorite in calcium silicate board and its influence factors under autoclaved curing. Constr. Build. Mater..

[47] Zou J.J., Guo C.B., Zhou X.Q., Sun Y.J., Yang Z. (2018). Sorption capacity and mechanism of Cr^3+^ on tobermorite derived from fly ash acid residue and carbide slag. Colloid Surf. A-Physicochem. Eng. Asp..

[48] Cai Y.C., Li C.L., Wu D., Wang W., Tan F.T., Wang X.Y., Wong P.K., Qiao X.L. (2017). Highly active MgO nanoparticles for simultaneous bacterial inactivation and heavy metal removal from aqueous solution. Chem. Eng. J..

[49] Gordienko P.S., Yarusova S.B., Suponina A.P., Yakimenko L.V. (2013). Sorption of Cd^2+^ ions by silicate materials of different origins. Russ. J. Gen. Chem+..

[50] Li Y., Liang Y.Q., Mao X.M., Li H. (2022). Efficient removal of Cu(II) from an aqueous solution using a novel chitosan assisted EDTA-intercalated hydrotalcite-like compound composite: Preparation, characterization, and adsorption mechanism. Chem. Eng. J..

[51] Qin J., Cui C., Cui X.Y., Hussain A., Yang C.M., Yang S.H. (2015). Recycling of lime mud and fly ash for fabrication of anorthite ceramic at low sintering temperature. Ceram. Int..

[52] Qin J., Cui C., Cui X.Y., Hussain A., Yang C.M. (2015). Preparation and characterization of ceramsites from lime mud and coal fly ash. Constr. Build. Mater..

[53] Guo X.L., Shi H.S. (2017). Microstructure and heavy metal adsorption mechanisms of hydrothermally synthesized Al-substituted tobermorite. Mater. Struct..

[54] Yang X.L. (2014). Reactivity Waster AAC and Preparation and Application of Silicate-Shell-Ceramsite. Ph.D. Thesis.

